# Association of Intrauterine and Early-Life Exposures with Diagnosis of Uterine Leiomyomata by 35 Years of Age in the Sister Study

**DOI:** 10.1289/ehp.0901423

**Published:** 2009-12-03

**Authors:** Aimee A. D’Aloisio, Donna D. Baird, Lisa A. DeRoo, Dale P. Sandler

**Affiliations:** Epidemiology Branch, National Institute of Environmental Health Sciences, National Institutes of Health, Department of Health and Human Services, Research Triangle Park, North Carolina, USA

**Keywords:** diabetes mellitus, diethylstilbestrol, early life, leiomyoma, phytoestrogens, pregnancy, prenatal exposure delayed effects, socioeconomic factors, soy formula

## Abstract

**Background:**

Early-life exposures to hormonally active compounds and other factors may affect later response to estrogen or progesterone and hence may influence development of uterine leiomyomata (fibroids).

**Objectives:**

We evaluated associations of *in utero* and early-life exposures, including soy formula, with self-report of physician-diagnosed fibroids by 35 years of age.

**Methods:**

Our study included 19,972 non-Hispanic white women who were 35–59 years of age when they enrolled in the Sister Study in 2003–2007. We estimated risk ratios (RRs) and 95% confidence intervals (CIs) using log-binomial regression models for fibroid associations with adjustment for participant’s age and education, maternal age at participant’s birth, birth order, and childhood family income.

**Results:**

Greater risk of early fibroid diagnosis was associated with soy formula during infancy (RR = 1.25; 95% CI, 0.97–1.61), maternal prepregnancy diabetes (RR = 2.05; 95% CI, 1.16–3.63), low childhood socioeconomic status (RR = 1.28; 95% CI, 1.01–1.63), and gestational age at birth (RR = 1.64; 95% CI, 1.27–2.13, for being born at least 1 month early). *In utero* diethylstilbestrol (DES) exposure was also associated with early fibroid diagnosis (RR = 1.42; 95% CI, 1.13–1.80), but this association was driven by women reporting probable rather than definite exposure.

**Conclusions:**

There are plausible biological pathways by which these early-life factors could promote fibroid pathogenesis. This is the first epidemiologic study to evaluate such exposures, with the exception of *in utero* DES, in relation to fibroid risk, and replication of findings in other populations is needed.

Uterine leiomyomata (fibroids) are benign smooth-muscle tumors, which are associated with pelvic pain, heavy bleeding, and reproductive problems (Stewart 2001), and this accounts for their being the most common indication for hysterectomies in the United States (Farquhar and Steiner 2002). The National Institute of Environmental Health Sciences (NIEHS) Uterine Fibroid Study, which used ultrasound screening to detect fibroids in participants, estimated that the risk of fibroids by 50 years of age exceeds 80% among African Americans and is nearly 70% among Caucasians (Baird et al. 2003). However, prevalence estimates based on clinically evident diagnoses have been approximately 25% (Stewart 2001). Age in premenopausal women and African-American race/ethnicity have been the most consistently reported risk factors (Baird et al. 2003; Faerstein et al. 2001; Marshall et al. 1997).

Both estrogen and progesterone have been implicated in fibroid pathogenesis, although the mechanisms by which these hormones act have not been elucidated (Marsh and Bulun 2006). Early-life and childhood exposures can affect uterine development and women’s response to estrogen or progesterone later in life and thus may influence fibroid development (Baird 2004). For instance, infants fed with soy formula are exposed to higher levels per unit body weight of estrogenic isoflavones than are adults consuming soy-based foods (Setchell et al. 1997). Although data in humans on long-term outcomes are limited, one small study has shown greater side effects of menstruation among women fed soy formula (Strom et al. 2001). Other early-life and childhood exposures also have not been previously investigated in relation to fibroids, except for *in utero* diethylstilbestrol (DES) (Baird and Newbold 2005; Wise et al. 2005a), age at menarche (Marshall et al. 1998; Wise et al. 2004), and childhood obesity (Terry et al. 2007).

We examined whether *in utero*, early-life, and childhood exposures were associated with self-report of fibroid diagnosis by 35 years of age among non-Hispanic white participants in the NIEHS Sister Study. Specifically, we evaluated the following participant factors: birth weight, gestational age at birth, birth order, being from a singleton or multiple birth, breast milk or soy formula consumption during infancy, relative height and weight during childhood, age at menarche; and the following childhood socioeconomic factors: maternal education, highest level of education in the household, food insecurity, and relative family income. In addition, we evaluated maternal factors related to the pregnancy with the participant: age at birth, living and working on a farm, smoking during pregnancy, prepregnancy diabetes, DES use, and complications of pregnancy (preeclampsia, pregnancy-related hypertension, and gestational diabetes).

## Materials and Methods

### Study population

The NIEHS Sister Study (NIEHS 2010) is a prospective cohort study that evaluates environmental and genetic risk factors for breast cancer and other end points in approximately 50,000 U.S. and Puerto Rican volunteer women 35–74 years of age. Eligibility criteria also included no previous diagnosis of breast cancer and a full or half-sister who was diagnosed with breast cancer. Study materials were available in English and Spanish. Recruitment for the Sister Study began in August 2003 with a vanguard group of women from four U.S. cities. The Sister Study opened nationally in October 2004 and closed in March 2009. Of 62,812 eligible women who agreed to enroll, 50,884 completed all baseline enrollment activities (fully enrolled) by 31 July 2009. The geographic distribution of participants for U.S. census regions and Puerto Rico is approximately 22% (West), 27% (Midwest), 17% (Northeast), 33% (South), and 2% (Puerto Rico).

Participants were mailed kits containing three questionnaires for self-completion (diet, family history, and use of personal care products), written consent documents, and support information for telephone interviews and home visits. A prepaid phone card was included to encourage participants to contact their mother or other relatives about early-life events and family medical history. Home visits included measurement of participants’ height and weight and retrieval of completed questionnaires. Participants completed a computer-assisted telephone interview to collect information on known and suspected risk factors for breast cancer and other end points. The Sister Study was approved by the Institutional Review Board of the NIEHS, National Institutes of Health, and all participants provided their informed consent.

Baseline data were available for 32,071 women who completed their interview and home visit by 21 September 2007. We restricted our analysis to 20,061 non-Hispanic whites who were 35–59 years of age when fully enrolled into the Sister Study. We excluded women > 59 years of age because of the likelihood of secular differences in use of ultrasounds for fibroid diagnoses and decreased ability to get information from their mothers about early-life events. Because of racial/ethnic differences in fibroid risk and related morbidity (Baird et al. 2003; Faerstein et al. 2001; Marshall et al. 1997; Wise et al. 2005b), we restricted our study population to non-Hispanic whites because we did not have sufficient numbers to separately evaluate other races/ethnicities at the time of our analysis. The present analysis includes 19,972 women after excluding 43 women who were missing data on fibroid status, 40 with missing age at diagnosis, and 6 who reported childhood or adolescent ages at diagnosis (< 15 years). Although the Sister Study is a prospective cohort study, our analysis is based on retrospectively collected information on factors that occurred before the diagnosis of fibroids.

### Fibroid assessment

During the baseline interview, women reported whether they were ever told by a doctor or health professional that they had uterine fibroids and their age at time of diagnosis. Because ultrasound screening has shown that the prevalence of fibroids increases rapidly after 35 years of age in U.S. white women (Baird et al. 2003), many older women will have undiagnosed fibroids. Therefore, we limited our case group to those who reported a fibroid diagnosis by 35 years of age to reduce disease misclassification in noncases.

### Exposure and covariate assessment

We used self-administered family history questionnaires to assess most intrauterine and early-life exposures: birth weight (pounds/ounces), gestational age at birth (categories), singleton or multiple birth, breast milk or soy formula consumption during infancy; and maternal exposures: age at birth, living and working on a farm, smoking during pregnancy, prepregnancy diabetes, DES use, and complications of pregnancy (preeclampsia, pregnancy-related hypertension, and gestational diabetes). Except for questions on gestational age, birth weight, singleton or multiple birth, and maternal age at birth, the response categories were “definitely,” “probably,” “probably not,” and “definitely not” to allow for possible uncertainty about early-life events. Questionnaires also assessed the duration that participants were fed with breast milk or soy formula and whether they were fed with soy formula during the first 2 months of life. However, we did not evaluate duration of breast-feeding or soy formula in relation to fibroids because of a considerable proportion of missing responses among women who reported being breast-fed (> 20%) or fed soy formula (> 30%). For analyses, we did not consider mothers as having gestational diabetes if they were reported to have diabetes before pregnancy. If participants reported that their mothers had both preeclampsia and pregnancy-related hypertension, we considered their mothers as having preeclampsia in analyses, and we considered mothers as having pregnancy-related hypertension only if preeclampsia was not also reported.

Birth order was estimated from birth dates of brothers reported on the family history questionnaires and sisters reported during the computer-assisted telephone interviews. We included all full siblings and half-siblings who shared the same mother for defining birth order. Childhood exposure data—maternal education and maximum level of education for parents or guardian in the household when participant was 13 years of age, relative family income based on self-reported categories (poor, low, middle, or well off), not enough to eat at times during childhood, age at menarche, and height and weight relative to peers at age 10—were collected during the telephone interview. Participant’s age, highest level of education, smoking status, alcohol intake, parity, and menopausal status were also assessed during the telephone interview, and body mass index was calculated using weight and height measured at the home visit.

### Statistical analyses

All statistical analyses were conducted using SAS (version 9.1; SAS Institute Inc., Cary, NC). We used log-binomial regression to estimate risk ratios (RRs) with 95% confidence intervals (CIs) for associations between each of the intrauterine, early-life, and childhood exposures and fibroid diagnosis by 35 years of age. For each of the relevant exposures, we combined women who reported definite or probable exposure for estimation of RRs and considered women unexposed if they reported probably not or definitely not. We later performed sensitivity analyses in which we estimated associations separately for women who reported definite exposure and those who reported probable exposure. We estimated associations with fibroids within the following categories of birth weight: < 2,500 g, 2,500–2,999 g, 3,000–3,499 g, 3,500–3,999 g, and ≥ 4,000 g. For maternal age, we initially examined associations with 5-year age categories and then further combined categories that were associated with a similar risk of fibroids. Likewise, we initially explored fibroid associations across all categories of birth order and maternal and household education, and then combined categories with similar fibroid risk for reporting of associations. Although approximately 40% of women did not report gestational age at birth, we evaluated its association with fibroids, categorizing women as having been born at least 1 month early, 2–4 weeks early, and not early (< 2 weeks before due date, on time, or late). Birth weight analyses were repeated with exclusion of women who reported being born at least 1 month early to further our understanding of birth weight and gestational age associations. We estimated RRs that were adjusted for participant factors (participant’s age and education) that may affect reporting of fibroids and exposures. We also adjusted RRs for early-life factors (birth order, maternal age at birth, and family income) that may be associated with other exposures and fibroids but were not considered to be on the causal pathway. The sample sizes for age-adjusted and fully adjusted models shown in tables differ. Although these differences are only about 2%, we repeated the age-adjusted analyses for the 19,531 women with complete data on variables included in the fully adjusted models and verified that results were similar to those for the complete sample of 19,972 women (data not shown).

## Results

The prevalence of self-reported fibroids diagnosed at any age was 25% (data not shown), with 8% of women reporting early diagnosis by 35 years of age (Table 1). Women reporting early diagnosis of fibroids (cases) were slightly less likely than women without early diagnosis (noncases) to be younger (35–44 years, 16% vs. 20%) or to have a bachelor’s or graduate degree (53% vs. 57%). As expected, cases were more likely than noncases to report being surgically menopausal at enrollment (48% vs. 21%). We also found that a greater proportion of cases reported having a hysterectomy by 35 years of age than noncases (21% vs. 5%) (data not shown). We had expected a greater proportion of cases than noncases to have been nulliparous at 35 years of age, but proportions were similar (25% vs. 26%). However, as suggested by Baird and Dunson (2003), we further restricted our evaluation of live or still births to those that occurred between 25 and 35 years of age and found that cases were more likely to report having no live or still births during these ages than were noncases (45% vs. 39%) (data not shown).

### Early-life factors

Five of the 14 early-life factors we examined were associated with more than a 20% increase in risk of early fibroid diagnosis (DES, prepregnancy diabetes, gestational diabetes, soy formula, and gestational age at birth) (Table 2). We noted the strongest association with fibroids for maternal diabetes before pregnancy (fully adjusted RR = 2.05; 95% CI, 1.16–3.63). We found an increased risk of fibroids in association with being fed soy formula within the first 2 months of life (adjusted RR = 1.25; 95% CI, 0.90–1.73), which was similar to that for any soy formula use (adjusted RR = 1.25; 95% CI, 0.97–1.61). Although only 60% of women reported gestational age at birth, we found a strong association with being born at least 1 month early (adjusted RR = 1.64; 95% CI, 1.27–2.13). Weak associations of at least a 10% increase in risk of fibroids were also noted for being firstborn, low birth weight (< 2,500 g), and reporting maternal pregnancy-related hypertension or preeclampsia. However, when we excluded women who reported being born at least 1 month early, there was no association with low birth weight (adjusted RR = 0.99; 95% CI, 0.79–1.25). Maternal age at participant’s birth also was weakly associated with increased fibroid risk. However, when we excluded firstborn women from analyses, the risk of fibroids was no longer elevated for having younger mothers (< 20 years), although the association for having older mothers (≥ 40 years) remained (data not shown). Having a mother who worked and lived on a farm or smoked during her pregnancy, being from a multiple birth, and having been breast-fed were not associated with fibroids.

Overall, the age-adjusted and fully adjusted RR estimates for the 14 evaluated exposures were similar, although the association between young maternal age (< 20 years) and fibroids was attenuated in the fully adjusted model. After repeating our analyses by estimating fibroid associations separately for women who reported “definitely” and those who reported “probably” for relevant early-life exposures, reporting of probable *in utero* exposure to DES (fully adjusted RR = 2.07; 95% CI, 1.53–2.80) and gestational diabetes (fully adjusted RR = 1.88; 95% CI, 0.94–3.74) were associated with fibroids, but there were no associations with definite *in utero* exposure to DES and gestational diabetes (Table 3).

### Childhood factors

Childhood socioeconomic factors, including less than high school for highest level of household education, not enough to eat at times during childhood, and being poor in childhood, were associated with fibroids, with the strongest association for being poor (RR = 1.24; 95% CI, 0.99–1.55) (Table 4). Because these socioeconomic factors are correlated, we evaluated whether fibroid associations were stronger with having multiple factors indicating low socioeconomic status. Having two or three of these childhood socioeconomic factors resulted in a slightly stronger association with fibroids (RR = 1.28; 95% CI, 1.01–1.63) than having only one of these factors (RR = 1.17; 95% CI, 1.01–1.35). As expected from previous studies (Marshall et al. 1998; Wise et al. 2004), earlier age at menarche was associated with greater risk of early fibroid diagnosis. Taller height and heavier weight at 10 years of age relative to peers were only weakly associated with fibroids.

## Discussion

We estimated an increased risk of early fibroid diagnosis in association with being fed soy formula during infancy, having a mother with prepregnancy diabetes, being born at least 1 month early, and reporting factors indicating low socioeconomic status during childhood (low household education, being poor, and not having enough to eat). We also noted associations with fibroids for having a mother with gestational diabetes and DES use during pregnancy, although these associations were restricted to reporting probable rather than definite exposure.

The association with soy formula is of interest given the estrogenic isoflavones found in soy products. Infants fed soy formula are exposed to isoflavone levels that are more than five times higher than typical levels for adults consuming soy-based foods (Setchell et al. 1997). Genistin is the naturally occurring isoflavone predominantly contained in soy formula, which can easily be hydrolyzed in the gut to the estrogenically active form of the compound, genistein, based on reporting of high plasma (Setchell et al. 1997) and urinary (Cao et al. 2009; Hoey et al. 2004) concentrations of genistein in infants fed soy formula. The increased risk of fibroids in association with being fed soy formula within the first 2 months of life, which may include the time period most sensitive to genistein exposure, was similar to the association with soy formula at any time during infancy.

Genistein has been extensively investigated in laboratory animals. In particular, neonatal treatment of mice with genistein has been associated with development of uterine adenocarcinoma (Newbold et al. 2001), abnormalities with mammary gland development and differences with mammary gland levels of estrogen and progesterone receptors (Padilla-Banks et al. 2006), alterations with estrous cycles and reduced fertility (Jefferson et al. 2005, 2007), and early reproductive senescence (Jefferson et al. 2005). However, studies with adult outcomes in humans have been lacking, except for one small study reporting longer duration and increased pain with menstrual bleeding for women who were fed soy formula (Strom et al. 2001). This finding is interesting given that pelvic pain and heavy bleeding are the most common symptoms of fibroids.

Our strongest association with fibroids was for maternal prepregnancy diabetes. Prenatal exposure to diabetes has also been associated with increased risk of obesity, abnormal glucose tolerance, and type 2 diabetes in adulthood (Dabelea 2007; Fetita et al. 2006). However, this is the first study to evaluate whether *in utero* exposure to maternal diabetes affects fibroid risk in early adulthood. One hypothetical mechanism by which *in utero* exposure to diabetes would affect later fibroid pathogenesis is the alteration of methylation patterns in regions that affect expression of relevant genes. In particular, expression of imprinted genes is especially sensitive to changes in methylation patterns (Thompson et al. 2001; Waterland and Jirtle 2004). Two studies in mice have reported that exposure of fetuses to induced maternal diabetes and of embryos before implantation to *in vitro* insulin affected expression of two imprinted neighboring genes, *H19* and *IGF2*, by altering their methylation status and resulted in changes in fetal development and birth weight (Shao et al. 2007, 2008). Seven microarray studies reviewed by Arslan et al. (2005) reported at least a 2-fold increase in *IGF2* expression in fibroids relative to normal myometrium.

Using the animal model of fibroids, the Eker rat, Cook et al. (2007) found an association between early exposure to DES and later fibroid development. Consistent with the animal model, Baird and Newbold (2005) reported a positive association of self-reported *in utero* DES exposure with fibroid diagnosis based on ultrasound assessment within the NIEHS Uterine Fibroid Study. However, Wise et al. (2005a) reported no association between maternal DES use and fibroids based on medically documented DES exposure and surgical fibroid cases. Given our inconsistent associations for women who report definite versus probable *in utero* DES exposure, conclusions from our study are unclear.

We found a consistent association of fibroids with three indicators of low socioeconomic status during childhood (low household education, food insecurity, and poverty). These factors may influence development of fibroids through changes in methylation patterns in childhood that persist and affect gene expression as adults. Plausibility of this hypothesis is based on animal and human data on early-life neglect or abuse. Different methylation patterns within the hippocampus of adult rats were detected based on whether they received maternal care early in life (Weaver et al. 2004). In a small sample of adult men who committed suicide, methylation patterns in similar genes in the hippocampus varied based on whether they were abused during childhood (McGowan et al. 2009). Whether being exposed to low socioeconomic conditions during childhood can affect methylation patterns in genes relevant to fibroid pathogenesis needs further investigation.

We also found a strong association with fibroids for being born at least 1 month before mother’s due date. A weak association with low birth weight was no longer present after excluding women who were born at least 1 month early. Because levels of estrogen and progesterone rise throughout pregnancy, one hypothesis is that women who are born early are deprived of the estrogen needed for full differentiation of their reproductive system (Trotter and Pohlandt 2000). Our analyses were limited by having only 60% of women who reported gestational age at birth. Missing data may be related to the true values of gestational age or other birth-related variables, but results were similar when we repeated the analyses assuming women missing gestational age data were not born ≥ 2 weeks early (data not shown).

Selection bias is a potential limitation for other exposures as well, given that the proportion of missing values was as high as 20%. There were slight differences in the proportion with fibroids based on whether exposures were missing, with generally more women reporting fibroids among those with missing responses. Based on the assumption that women with missing data for rare exposures (preeclampsia, pregnancy-related hypertension, soy formula, DES use, prepregnancy diabetes, and gestational diabetes) were likely to be unexposed, we repeated analyses in which we considered women with missing values for these factors as unexposed. However, changes in RR estimates were minimal (data not shown).

There is also the potential for misclassification of exposures given that women were reporting exposures during infancy and related to their mother’s pregnancy. However, women were provided phone cards to encourage them to ask these questions directly of their mothers, and we excluded older women (> 59 years) who would be less likely to have living mothers to ask about these exposures. In addition, response categories for many of the exposures included options of “definite” and “probable” that allowed for uncertainty in reporting. Associations with fibroids were generally consistent for definite and probable exposure, except associations with *in utero* DES exposure and maternal gestational diabetes, for which associations were much stronger with reporting probable exposure. Because none of these exposures is known to be related to fibroids, exposure misclassification would likely be nondifferential, which would generally result in RR estimates biased toward the null.

Our assessment of fibroid diagnoses was based exclusively on self-report. However, because fibroid incidence increases strongly with age, we only considered diagnoses by 35 years of age to reduce misclassification in the noncase group. Our estimated risk of 8% for early diagnosis of fibroids was similar to the risk of 11% for self-reported fibroids by 35 years of age in white women (35–49 years of age) from the NIEHS Uterine Fibroid Study (Baird DD, unpublished observations). We also reported a substantially greater proportion of women with early diagnosis of fibroids having hysterectomies compared with women without early diagnosis, which suggests that many of the women with fibroid diagnoses included in our case definition had fibroid-related morbidity. However, we do not have information on fibroid-related symptoms at time of diagnosis. We also excluded older women from our analyses because of possible secular differences in use of ultrasounds for fibroid diagnoses. Because studied factors may also be related to fibroids diagnosed later in life, including women with fibroids diagnosed after 35 years of age as noncases may have resulted in an underestimation of RRs. However, repeating our analyses after exclusion of women with later diagnoses of fibroids (> 35 years) from the noncase group did not affect RR estimates with early diagnosis of fibroids for four of our main findings (maternal prepregnancy diabetes, soy formula, low childhood socioeconomic status, and being born at least 1 month early (data not shown).

Strengths of this study include a large sample size, which allowed us to examine associations with rare intrauterine and early-life exposures. We adjusted for factors that may affect recall of exposures, including participant’s age and education. In addition, despite the potential for misclassification bias from self-reported exposure information and fibroid diagnoses, we observed expected associations between specific factors, including a positive association between early age at menarche and fibroids and an increased reporting of maternal preeclampsia among firstborn women and those from a multiple birth (data not shown).

## Conclusions

Our study suggests that being fed with soy formula during infancy, having a mother with prepregnancy diabetes, being born at least 1 month early, and growing up with low socioeconomic conditions may increase the development of fibroids in early adulthood. This is the first study to explore these early-life and childhood factors in relation to the risk of fibroids. There are plausible biological mechanisms by which these factors could affect uterine physiology later in life and thus increase risk of fibroid development. Replication of findings in other populations including higher risk groups such as African Americans is needed.

## Correction

In Table 1, the values for body mass index were incorrect in the manuscript originally published online. They have been corrected here.

## Figures and Tables

**Table 1:** Participant characteristics [*n* (%)] by uterine fibroids status of non-Hispanic whites, 35–59 years of age, in the Sister Study, 2003–2007 (*n* = 19,972).^a^

Characteristic	Cases	Noncases
Total	1,526	18,446
Age (years)		
35–44	241 (15.8)	3,712 (20.1)
45–59	1,285 (84.2)	14,734 (79.9)
Highest level of education^b^		
High school or less^c^	201 (13.2)	2,159 (11.7)
Some college/vocational school	519 (34.0)	5,809 (31.5)
Bachelor’s degree	403 (26.4)	5,706 (30.9)
Graduate degree	403 (26.4)	4,771 (25.9)
Smoking status^b^		
Current	195 (12.8)	2,105 (11.4)
Former	496 (32.5)	6,125 (33.2)
Never	835 (54.7)	10,213 (55.4)
Alcohol intake (drinks per week)^b^		
0	242 (15.9)	2,529 (13.7)
0.01–0.49	446 (29.3)	5,189 (28.2)
0.50–2.00	341 (22.4)	4,257 (23.1)
2.01–6.99	305 (20.0)	3,821 (20.7)
≥ 7.00	190 (12.5)	2,637 (14.3)
Body mass index^b^		
< 25	614 (40.4)	8,217 (44.8)
25–29.9	479 (31.5)	5,421 (29.5)
≥ 30	428 (28.1)	4,720 (25.7)
Parity^b^,^d^		
0	381 (25.0)	4,883 (26.5)
1	297 (19.5)	3,049 (16.5)
2	553 (36.3)	6,751 (36.6)
≥ 3	293 (19.2)	3,754 (20.4)
Menopausal status^b^		
Premenopausal	449 (29.4)	8,733 (47.4)
Natural	346 (22.7)	5,793 (31.4)
Surgical^e^	731 (47.9)	3,914 (21.2)

a Cases were self-reported diagnosis of fibroids at ≤ 35 years of age, excluding 89 women missing fibroid status or age at diagnosis or who reported childhood or adolescent ages at diagnosis (< 15 years).

b Number does not sum to total because of missing data.

c Less than high school: fibroids (*n* = 13), no fibroids (*n* = 79).

d Total number of live and still births by 35 years of age.

e Includes women with hysterectomy or endometrial ablation in which actual timing of hormonal transition is unknown.

**Table 2:** RRs for associations of uterine fibroids with *in utero* and early-life exposures among non-Hispanic whites, 35–59 years of age, in the Sister Study, 2003–2007.^a^

	Age adjusted (*n* = 19,972)^b^				Fully adjusted (*n* = 19,531)^b^			
Exposure	*n*	No. of cases	RR	95% CI	*n*	No. of cases	RR	95% CI
Maternal pregnancy factors								
Worked and lived on farm^c^,^d^								
Yes	1,611	134	1.08	0.91–1.28	1,588	132	1.02	0.86–1.22
No^e^	16,823	1,275	1.00		16,639	1,262	1.00	

Smoking^c^,^d^								
Yes	6,987	533	1.01	0.91–1.12	6,893	529	1.02	0.92–1.13
No	12,142	916	1.00		12,025	905	1.00	

DES use^c^,^d^								
Yes	658	69	1.40	1.11–1.76	643	68	1.42	1.13–1.80
No	16,723	1,234	1.00		16,563	1,224	1.00	

Prepregnancy diabetes^c^,^d^								
Yes	64	10	2.08	1.18–3.69	64	10	2.05	1.16–3.63
No	19,541	1,484	1.00		19,329	1,468	1.00	

Gestational diabetes^c^,^d^								
Yes^f^	101	9	1.25	0.67–2.34	101	9	1.28	0.68–2.38
No	18,283	1,373	1.00		18,101	1,359	1.00	

Preeclampsia/eclampsia^c^,^d^								
Yes	394	37	1.26	0.92–1.71	390	36	1.20	0.87–1.64
No	17,247	1,283	1.00		17,077	1,270	1.00	

Pregnancy-related hypertension^c^,^d^								
Yes^g^	243	20	1.11	0.73–1.70	243	20	1.12	0.73–1.71
No	16,459	1,238	1.00		16,299	1,226	1.00	

Maternal age at birth (years)^c^								
< 20	688	68	1.31	1.04–1.66	676	67	1.19	0.92–1.53
20–24	4,334	345	1.06	0.94–1.20	4,310	342	1.01	0.89–1.15
25–34	10,801	806	1.00		10,764	803	1.00	
35–39	2,787	195	0.95	0.82–1.11	2,780	195	0.96	0.82–1.11
≥ 40	1,003	83	1.14	0.91–1.41	1,001	83	1.14	0.92–1.42

Participant factors								
Firstborn^c^								
Yes	3,850	326	1.12	0.99–1.25	3,834	326	1.11	0.97–1.27
No	15,842	1,173	1.00		15,697	1,164	1.00	

Birth weight (g)^c^								
< 2,500	1,382	120	1.13	0.93–1.37	1,361	118	1.12	0.92–1.36
2,500–2,999	3,086	244	1.04	0.90–1.21	3,050	242	1.04	0.89–1.20
3,000–3,499	6,191	471	1.00		6,141	466	1.00	
3,500–3,999	3,928	281	0.94	0.82–1.09	3,897	278	0.94	0.82–1.09
≥ 4,000	1,364	108	1.05	0.86–1.28	1,354	107	1.05	0.86–1.29

Gestational age at birth^c^								
Born ≥ 1 month early	464	54	1.63	1.26–2.11	459	54	1.64	1.27–2.13
Born 2–4 weeks early	1,017	77	1.08	0.86–1.35	1,010	76	1.08	0.86–1.36
Not born ≥ 2 weeks early	10,129	716	1.00		10,031	706	1.00	

Multiple birth^c^								
Yes	692	49	0.93	0.71–1.22	679	48	0.93	0.70–1.22
No	19,037	1,454	1.00		18,825	1,439	1.00	

Fed breast milk^c^,^d^								
Ever	7,240	538	0.95	0.86–1.05	7,167	526	0.93	0.84–1.03
None	11,543	890	1.00		11,421	887	1.00	

Fed soy formula^c^,^d^								
Ever	645	60	1.28	1.00–1.64	641	58	1.25	0.97–1.61
None	16,180	1,212	1.00		16,012	1,201	1.00	

Fed soy formula, age ≤ 2 months^c^								
Yes	386	34	1.22	0.88–1.69	384	34	1.25	0.90–1.73
No	16,387	1,226	1.00		16,215	1,215	1.00	

a Fibroids status based on self-reported diagnosis at ≤ 35 years of age, excluding 89 women missing fibroid status or age at diagnosis or who reported childhood or adolescent ages at diagnosis (< 15 years).

b Each age-adjusted model included participant’s age; each fully adjusted model included the following covariates: participant’s age and education, maternal age, firstborn status, and childhood family income.

c *n* does not sum to total because of missing data. Percent with missing data was as high as 20%, with the exception of 40% with missing gestational age at birth.

d Yes or ever represents reporting of definitely or probably. No or none represents reporting of probably not or definitely not.

e Excludes those reporting mothers who worked or lived on a farm during pregnancy (age adjusted, *n* = 956; fully adjusted, *n* = 949).

f Excludes those reporting mothers who definitely or probably had diabetes before pregnancy.

g Excludes those reporting mothers who definitely or probably had preeclampsia during pregnancy.

**Table 3:** RRs considering certainty of *in utero* and early-life exposures in uterine fibroid associations among non-Hispanic whites, 35–59 years of age, in the Sister Study, 2003–2007.^a^

	Definite				Probable			
Exposure	*n*	No. of cases	aRR^b^	95% CI	*n*	No. of cases	aRR^b^	95% CI
Worked and lived on farm^c^	970	80	1.02	0.82–1.27	618	52	1.04	0.79–1.35
Smoking during pregnancy	5,040	368	0.97	0.87–1.10	1,853	161	1.14	0.97–1.34
DES use	404	31	1.04	0.74–1.46	239	37	2.07	1.53–2.80
Prepregnancy diabetes	29	5	2.26	1.02–5.01	35	5	1.88	0.83–4.23
Gestational diabetes^d^	50	2	0.60	0.15–2.33	51	7	1.88	0.94–3.74
Preeclampsia/eclampsia	248	22	1.15	0.77–1.72	142	14	1.28	0.78–2.11
Pregnancy-related hypertension^e^	115	11	1.30	0.74–2.28	128	9	0.96	0.51–1.80
Fed breast milk	6,356	462	0.92	0.83–1.03	811	64	0.99	0.77–1.26
Fed soy formula	481	46	1.32	1.00–1.75	160	12	1.03	0.60–1.78

aRR, adjusted risk ratio.

a Fibroids status based on self-report of diagnosis at ≤ 35 years of age, excluding 89 women missing fibroid status or age at diagnosis or who reported childhood or adolescent ages at diagnosis (< 15 years).

b Each model included the following covariates: participant’s age and education, maternal age, firstborn status, and childhood family income (*n* = 19,531). The reference group is women who reported probably not or definitely not for each exposure.

c Excludes from the reference group those reporting mothers who worked or lived on a farm (but not both) during pregnancy (*n* = 949).

d Excludes those reporting mothers who definitely or probably had diabetes before pregnancy.

e Excludes those reporting mothers who definitely or probably had preeclampsia during pregnancy.

**Table 4:** RRs for associations of uterine fibroids with childhood socioeconomic and developmental factors among non-Hispanic whites, 35–59 years of age, in the Sister Study, 2003–2007.^a^

	Age adjusted (*n* = 19,972)^b^				Fully adjusted (*n* = 19,531)^b^			
Exposure	*n*	No. of cases	RR	95% CI	*n*	No. of cases	RR	95% CI
Maternal education at participant age 13^c^								
< High school	2,988	250	1.10	0.96–1.25	2,925	246	1.05	0.91–1.21
≥ High school	16,339	1,220	1.00		15,986	1,188	1.00	

Maximum household education at participant age 13^c^								
< High school	1,876	173	1.21	1.04–1.41	1,833	171	1.15	0.98–1.36
≥ High school	18,007	1,347	1.00		17,618	1,313	1.00	

Family income^c^								
Poor	819	77	1.24	1.00–1.55	793	76	1.24	0.99–1.55
Low	4,279	345	1.07	0.95–1.20	4,192	342	1.07	0.95–1.21
Middle/well off	14,859	1,104	1.00		14,546	1,072	1.00	

Not enough to eat^c^								
Yes	1,400	129	1.22	1.02–1.44	1,346	128	1.16	0.96–1.40
No	18,567	1,396	1.00		18,180	1,361	1.00	

Age at menarche (years)^c^								
< 10	1,106	120	1.54	1.27–1.87	1,076	117	1.54	1.26–1.87
11	2,565	242	1.35	1.16–1.57	2,511	232	1.32	1.13–1.53
12	5,493	447	1.17	1.03–1.33	5,376	441	1.18	1.04–1.34
13	6,023	419	1.00		5,899	409	1.00	
14	2,760	178	0.93	0.79–1.10	2,692	173	0.93	0.78–1.10
≥ 15	2,006	119	0.86	0.70–1.04	1,958	117	0.86	0.71–1.05

Height relative to peers at age 10^c^								
Taller	5,962	486	1.09	0.97–1.22	5,830	474	1.10	0.98–1.23
Same	8,964	675	1.00		8,770	658	1.00	
Shorter	5,027	364	0.96	0.85–1.09	4,912	357	0.97	0.86–1.10

Weight relative to peers at age 10^c^								
Heavier	4,054	325	1.09	0.96–1.24	3,966	317	1.09	0.96–1.24
Same	9,162	676	1.00		8,960	660	1.00	
Lighter	6,725	521	1.05	0.94–1.17	6,575	509	1.05	0.94–1.17

a Fibroids status based on self-reported diagnosis at ≤ 35 years of age, excluding 89 women missing fibroid status or age at diagnosis or who reported childhood or adolescent ages at diagnosis (< 15 years).

b Each age-adjusted model included participant’s age; each fully adjusted model included the following covariates: participant’s age and education, maternal age, firstborn status, and childhood family income.

c *n* does not sum to total because of missing data.
