# Correction: Association of Intrauterine and Early-Life Exposures with Diagnosis  of Uterine Leiomyomata by 35 Years of Age in the Sister Study

**DOI:** 10.1289/ehp.0901423-erratum

**Published:** 2009-12-03

In Table 1, the values for body mass index were incorrect in the manuscript originally published online. They have been corrected here.

